# Expanded carrier screening in gamete donors of Venezuela

**DOI:** 10.5935/1518-0557.20170062

**Published:** 2017

**Authors:** Maria Teresa Urbina, Isaac Benjamin, Randolfo Medina, José Jiménez, Laura Trías, Jorge Lerner

**Affiliations:** 1 Unifertes Fertility Unit, Caracas, Venezuela.

**Keywords:** Expanded carrier screening, CF, SMA

## Abstract

**Objective:**

To discuss the implications of expanded genetic carrier screening for preconception purposes based on our practice.

**Methods:**

One hundred and forty-three potential gamete donors aged 20-32 years old (µ=24, 127 females and 16 males), signed informed consent forms and were selected according to the REDLARA guidelines. Blood or saliva samples were examined by one of these genetic carrier screening methods: Genzyme screening for Cystic Fibrosis (CF), Fragile X and Spinal Muscular Atrophy (SMA); Counsyl Universal panel or Recombine Carrier Map.

**Results:**

Genotyping results for all donors were analyzed; 41% (58/143) of donors were identified as carriers for at least one condition. We found a carrier frequency of 1/24 for CF, 1/72 for SMA and 0/120 for Fragile X syndrome. Among the high-impact most prevalent conditions in our study (Carrier Map group) were: 21-Hydroxilase-Deficient Congenital Nonclassical Adrenal Hyperplasia (1/8), Factor V deficiency (1/12), Hemochromatosis: Type 1: HFE Related (1/12), Short Chain Acyl-CoA (1/14) and MTHFR deficiency 1/3 (39%).

**Conclusions:**

The rate of gamete donors identified as carriers of at least one condition was 41%, which supports the offering of expanded carrier screening to our population. Studies in Latin American populations could help customize screening panels. The ART patient population has a unique opportunity to be offered expanded carrier screening and appropriate counseling, to make its best-informed decisions.

## INTRODUCTION

Gene-by-gene carrier screening has been available for years to patients with increased genetic risk and considering pregnancy (for example, Ashkenazi Jewish patients). Expanded genetic carrier screening (ECS) has been developed for many disorders, with low costs, enabling patients to consider several reproduction options. Guidelines regarding ethnicity and population-based genetic screening have been published by the American College of Obstetricians and Gynecologists (ACOG), the American College of Medical Genetics (ACMG), National Society of Genetic Counselors, Perinatal Quality Foundation and Society for Maternal-Fetal Medicine, Society of Obstetricians and Gynecologists of Canada, Canadian College of Medical Geneticists and others (ACOG, 2017a,b; Grody *et al.*, 2013; Edwards *et al.*, 2015; Wilson *et al.*, 2016). Guidelines for gamete carrier donors have also been published by the American Association of Reproductive Medicine (ASRM)/Society for Assisted Reproductive Technology (SART) (The Practice Committee of the American Society for Reproductive Medicine & the Practice Committee of the Society for Assisted Reproductive Technology, 2013) and Red Latino Americana de Reproducción Asistida (REDLARA, 2015) among others.

According to the ACOG, ethnic-specific, pan-ethnic, and ECS are acceptable strategies for preconception and prenatal carrier screening. ECS, enables patients to make informed decisions to plan pregnancy based on their personal values (ACOG, 2017a,b). The ACOG recommends offering ECS to every woman, regardless of ethnicity or family history (ACOG, 2017a,b). Other professional associations consider that ECS is still expensive in some countries, and produces anxiety to the patients if they cannot afford it. Because those are rare diseases and do not cause significant health impairments, screening could be unnecessary many times.

Objective: We discuss the implications of ECS for preconception purposes based on our own practice.

## MATERIALS AND METHODS

### Design: Retrospective study

One hundred and forty-three potential gamete donors aged 20-32 years old (µ=24, 127 females and 16 males), signed informed consent forms and were selected according to the REDLARA guidelines (REDLARA, 2015). Blood or saliva samples were examined by one of these genetic carrier screening methods:

- Genzyme: Cystic fibrosis (CF), Spine Muscular Atrophy (SMA) and Fragile X carrier tests.
- Counsyl Universal Panel: Targeted DNA mutation analysis to simultaneously determine the carrier status of an individual for 399 variants associated with 101 diseases (as of 2012).
- Recombine Carrier Map Panel: More than 981 mutations - 178 diseases (as of 2012). Panels changed over time; nowadays Carrier Map Expanded v3 is screening for 314 diseases, 302 genes, 2719 mutations (2017).

## RESULTS

Genotyping results for all donors were analyzed; 41% (58/143) of donors were identified as carriers of at least one condition. We found a carrier frequency of 6/143 (1/24) for CF, 2/143 (1/72) for SMA and 0/120 for Fragile X syndrome. Ninety-six donors were analyzed with ECS by:

COUNSYL (n=24): 7 donors with 1 detected mutation: 2 with CF, 2 with Glycogen Storage Disease Type V, Alpha-1 antitrypsin deficiency, Pompe disease, Achromatopsia.

RECOMBINE (n=72): 39 were positive for at least one condition, 38 of them were a carrier of at least one high impact disease. The average carrier burden of recessive conditions was 2.1 mutations per donor (1-6). The most prevalent condition was the 5, 10 methylenetetrahydrofolate reductase (MTHRF) deficiency: 39% (28/72) of donors.

Results are shown in Table 1.

**Table 1:** Number of gamete donors per condition.

Number of gamete donors per condition (n=143)				Disease severity*			
	Genzyme 2010 (n=47)	Counsyl 2012 (n=24)	Recombine 2012 (n=72)	H	M	T	X
**Cystic fibrosis**	4	2	0	x		x	
**Fragile X syndrome**	0		0	x			x
**Spine muscular atrophy**	2	0	0	x			
**21-Hydroxilase-Deficient Congenital Nonclasical Adrenal Hyperplasia**			9		x	x	
Achromatopsia		1	1				
Alpha-1-antitrypsin deficiency		1	2		x		
Autosomal Recessive Kidney Disease			1				
Autosomal Recessive Polycystic Kidney Disease		0	2	x			
Bartter Syndrome: Type 4A			1	x		x	
Biotinidase Deficiency		1	3	x		x	
Carnitine Palmitoyltransferase II Deficiency		0	1	x		x	
Cystinuria: No Type 1			1	x		x	
Duarte Galactosemia			2				
Factor V Leiden thrombophilia deficiency			2				
**Factor V deficiency**			6				
Familial Mediterranean fever		0	2	x		x	
Glucose-6-Phosphate Dehydrogenase Deficiency			2		x	x	x
Glutamic Acidemia: Type I			1	x		x	
Glycogen Storage Disease Type V		2	0		x		
Glycogen Storage Disease: Type IV			1	x			
**Hemochromatosis: Type 1: HFE Related**			6	x		x	
Homocystinuria Caused by CBS Deficiency: B6 Responsive		0	3	x		x	
Leber Amaurosis			2	x			
**MTHFR Deficiency**			28	x		x	
Nonsyndromic hearing loss and Deafness			2	x			
Phenylalanine Hydroxylase Deficiency			1	x		x	
POLG Related Disorders			1	x			
Pompe Disease		1					
Pseudocholinesterase Deficiency		0	1		x	x	
**Short Chain Acyl-CoA**		0	7		x	x	
Sulfate Transporter-related Osteochondrodysplasia		0	1	x			
Tyrosinemia: Type 1		0	1	x			
Sickle Cell Anemia			1	x			

* **According to Recombine classification:**

**H:** High impact, significant effect on life expectancy and quality of life.

**M:** Moderate impact, do not affect life expectancy but can affect the quality of life.

**T:** Treatment benefits, can lessen the impact of these diseases, especially with early intervention

**X:** X-linked diseases are passed down by female carriers. Carriers may have symptoms.

Blank cells indicate that condition was not screened for.

## DISCUSSION

In 2010, a 51-year-old woman of Spanish In 2010, a 51-year-old woman of Spanish ancestry with 16 years of infertility, 15 IUI, one GIFT, and five ICSI cycles at several fertility clinics, had a baby conceived by IVF in our clinic using mixed European ancestry donor semen and oocytes from a 20-year-old donor of Italian and Spanish ancestry. Both donors had normal karyotypes. The donor semen screened negative as a carrier for CF and hemoglobinopathies.

At 10 months of age, the baby was unable to sit, stand, or crawl due to muscle weakness. The clinical diagnosis was SMA, a progressive neuromuscular disease that results in muscle weakness due to deterioration and loss of the anterior horn cells. It is inherited in an autosomal recessive manner due to mutations in the SMN1 gene. In the most common and severe form, SMA type 1, death occurs due to respiratory failure before 2 years of age. Carrier screening for SMA performed on both donors after baby diagnosis, confirmed that they carried a copy of the SMN1 deletion mutation. The child death occurred due to respiratory failure before reaching 2 years of age (Callum *et al*., 2010). The incidence of SMA is approximately 1 in 6,000 to 1 in 10,000 live births, and the disease is reported to be the leading genetic cause of infant death ('Committee Opinion No. 691: Carrier Screening for Genetic Conditions', 2017). It is a rare but high impact disease, due to the early onset of symptoms, significant suffering, and lack of available treatment (Callum *et al.*, 2010).

In 2010, when the IVF treatment was done, ASRM and REDLARA had established guidelines for minimum genetic screening for gamete donor applicants (1); however, SMA carrier screening was not included. General population carrier screening for SMA was controversial due to conflicting guidelines by the ACMG (2008) and by the ACOG at the time (2009) (Callum *et al.*, 2010).

Since gametes from an individual donor may be used by several recipients, there is an increased risk for autosomal recessive disease in the offspring due to multiple pairings, compared to the risk to offspring from a single couple. Therefore, the screening of genetic diseases for gamete donors is part of the necessary studies to reduce the risk to children born from gamete donor treatments. Hence, we decided to screen potential donors for common and severe disorders such as SMA, CF, and Fragile X (at Genzyme), routinely.

Several months later, Counsyl made the prospect of universal carrier testing feasible for the first time with a non-invasive, saliva-based assay, for more than 100 Mendelian diseases across all the main ethnic groups. The test was validated with a median of 147 positive and 525 negative samples per variant, demonstrating a multiplex assay which performance was compared with previous blood-based single-gene carrier tests. Consequently, we decided to replace the blood based gene-by-gene tests we were performing at Genzyme for the non-invasive, single test for hundreds of severe mutations at Counsyl (at the same cost) (Srinivasan *et al.*, 2013).

In 2012, Recombine offered Carrier Map Panel to screen for a much wider range of Mendelian diseases. Therefore, we decided to screen our gamete donors and patients with their panel. This test was designed with input from professional societies worldwide and covers the widest range of ancestries. A large proportion of the population in Venezuela are multi-ethnic, they do not know their ancestry, or identify with a particular ethnicity. Carrier Map includes conditions that are common in Latin America and includes all ACOG- and ACMG-recommended conditions plus over 300 others.

### Carrier rate and most prevalent conditions

The rate of donors identified as carriers for at least one condition was 41% (58/143). This carrier rate was surprisingly high compared with that reported for Hispanic population (9.8%) in a study on 400+ causal Mendelian variants from an ethnically diverse clinical sample of 23,453 individuals: carrier frequencies for self-reported ethnicity, ranged from 43.6% of Ashkenazi Jewish to 8.5% of East Asian (Lazarin *et al.*, 2013a).

Our early results showed a high prevalence for CF and SMA among donors, compared to the one reported for the Hispanic population, as our sample sizes increased, prevalence became more similar to those reported for Hispanic, Caucasian (ACOG, 2017a) and Argentinian (Quinteiro Retamar, 2015) populations. Venezuela is a multi-ethnic society (with Spanish, Italian, Portuguese, African, and Native American backgrounds). Our donors were not randomly selected; we chose donors physically similar to our patients. The two SMA carriers were sisters and had Italian and Spanish ancestry. These results stress the need to increase sample size screening (Table 2).

**Table 2:** Prevalence of relevant conditions comparison among our population and other reports.

	Genzyme. Early results (n=47)	All donors	General (ACOG 2017a)	Hispanic (ACOG 2017a)	Caucasian (ACOG 2017a )	Argentinian (Quinteiro Retamar *et al*., 2015)
**CF**	1:12	1:24 (6/143)	-	1:58	1:25	1:19
**SMA**	1:24	1:72 (2/143)	-	1:117	1:35	1:23
**Fragile X**	0:47	0:120	1:259	-	-	-

Disease severity is a key criterion to include a condition in the screening panels (Grody *et al.*, 2013; Lazarin *et al.*, 2014). Systematic classifications of disease severity of ECS have been proposed (Lazarin et al., 2014) (Table 1). The high-impact, most prevalent conditions in our study (Carrier Map group) were: 21-Hydroxilase-Deficient Congenital Nonclassical Adrenal Hyperplasia (1/8), Factor V deficiency (1/12), Hemochromatosis: Type 1: HFE Related (1/12), Short Chain Acyl-CoA (1/14) and MTHFR deficiency (1/3) (39%). It has been reported that approximately 20-40% of Caucasian or Hispanic individuals are heterozygous for MTHFR C677T in the US (Levin & Varga, 2016; Botto & Yang, 2000).

Most data available in the literature are for US Hispanics, information for Latin-American populations are scarce. To determine the diseases with the highest carrier frequency for Latin-American populations could help to customize screening panels.

### Benefits of ECS

Patients benefit from ECS by increasing their reproductive options: matching for carrier status, using gametes from an alternative donor, preimplantation genetic diagnosis, embryo donation, or even choosing a lifestyle without children. (Callum *et al.*, 2010) ECS would also reduce the risk for autosomal recessive diseases due to multiple pairings by gamete donation.

### Access

The low cost of ECS panels is one reason to support this practice. However, the actual costs of ECS need to be carefully examined. In Latin American countries, the cost of genetic carrier screening tests remains high and can result in inequitable distribution of reproductive rights of genetic testing and services. If patients believe that screening is a must, but it is not affordable to them, the situation could create unnecessary anxiety. Counseling costs should be considered.

Research and new developments will lower costs. In the long term, it could benefit social security programs because genetic disease prevention will diminish the healthcare burden to the state. Meanwhile, governments have the opportunity-and the responsibility-to close such gaps in reproductive health, providing coverage and access to ECS services.

### Psychosocial implications

ECS panels are often marketed directly to patients (Dondorp *et al.*, 2014). Geneticists worry that this is causing unnecessary testing and misconceptions. Lack of counseling could cause anxiety or alarm, or a wrong belief that ECS would warranty the birth of a healthy child.

Some conditions are rare or do not cause significant health impairment. Disorders to be included in ECS panels should be of a nature that most patients would consider having a prenatal diagnosis to facilitate making decisions around reproduction (Grody *et al.*, 2013). But, a study reported that ECS affected clinical decision making for patients in only 0.21% of cases: in 8 of 3,738 couples, both members were positive for the same genetic disorder or had a test result indicating that they were at risk of having an affected baby (Franasiak *et al.*, 2016).

Genetic testing may not reveal actionable information. If a woman tested positive for a condition, should we test her partner? What would we do with this info? Should we encourage taking the risk of CVS/amniocentesis or consider pregnancy termination based on positive carrier status in both partners? It is likely to cause anxiety for patients who don't understand why you tested them for these things if you cannot recommend doing anything further (Callum *et al.*, 2010; Grody *et al.*, 2013).

Psychosocial implications of ECS have been studied. A meta-analysis of long- and short-term effects demonstrated that anxiety was overruled by knowledge of reproductive options and may be calmed by counseling services. Guilt was significantly associated with individuals who knew their carrier status after they had an affected child ("survivor guilt") (Lazarin *et al.*, 2013b). Benefits of testing more often outweigh the risks, and the effects of not testing mean greater psychosocial risks (Lazarin *et al.*, 2013b).

Counselors should know the complexity of psychosocial experiences experienced by individuals and try to address misconceptions related to the carrier status and results warranty. Some patients manage anxiety through threat minimization as an active coping mechanism. Counselors should make certain that those who appear to be managing well, will not minimize their threat and therefore neglect to take preventive actions (Lewis *et al.*, 2011).

It is ethical to provide patients the opportunity to get the information about theirs or the donor genetic carrier status and appropriate counseling. Patients are able to evaluate complex information and take the best decisions based on their own values.

### Appropriate counseling and informed consent

Fertility centers have a duty to transfer the information to patients and to reduce the psychosocial impact of ECS (Grody *et al.*, 2013). To provide appropriate counseling to the patients, the education of health providers and psychologist is a need. Increased attention should be given to informing them about the test availability, diseases prevalence, reproductive options, residual risk, benefits, and limitations, of ECS for themselves and gamete donors. ACOG recommendations for counseling on genetic testing and communication of genetic test results has been published recently (ACOG, 2017c). ECS should only be performed after appropriate counseling and informed consent. From a clinical ethics perspective, informed consent is a communication process, and should not only be treated as a required form for the patient's signature. To prevent conflicts, it would aid implementation of generic informed consent as suggested by ACMG (Grody et al., 2013).

### Legal considerations for conflict prevention

There are two primary causes of potential civil actions against healthcare providers for conflicts resulting from healthcare: lack of informed consent, and violation of the standard of care. Because of it, we recommend appropriate patient counseling and informed consent but also the education of legal professionals, based on scientific evidence. Setting appropriate medico-legal standards regarding what can be expected from centers in terms of donor´s genetic testing and which test could be offered is recommended (Dondorp *et al.*, 2014).

Legal professionals must be made aware that screening panels change over time as science advances and tests for other disorders are developed. Guidelines regarding ethnicity and population-based genetic screening are frequently updated by genetic associations. Donors could only be evaluated by the current tests recommended at the time of the donation.

Legal professionals are urged to increase their knowledge of genetic screening terminology and consider that residual risk is always present. If one partner is found to be a carrier and the other has a negative result for the same condition, the probability that the couple will have an affected pregnancy is significantly reduced, but a negative screen does not eliminate risks to offspring. The partner is unlikely to have a mutation for the same disorder, but a residual risk persists. According to most guidelines, no further testing is needed; prenatal diagnosis is not indicated. Carrier screening will not identify all individuals who are at risk of the screened conditions and some cases can be the result of a new gene mutation. It is crucial to understand that ECS is a risk reducing rather than a risk eliminating opportunity (Srinivasan *et al.*, 2013).

## CONCLUSIONS

- The rate of gamete donors identified as carriers for at least one condition was 41%, which supports the provision of ECS to our population. More studies with ECS in Latin American populations could help to customize screening panels.
- The ART patient population has a unique opportunity to be offered ECS and appropriate counseling, to make its best-informed decisions.
- To provide appropriate counseling health care provider´s education is a need.
- To prevent conflicts, it would be beneficial to implement generic informed consent forms.
- Legal professionals are urged to increase their knowledge of genetic screening terminology, and consider that residual risks are always present. ECS is a risk reducing rather than a risk eliminating opportunity.
- We should promote universal access to ECS. This is just the beginning of genetic applications to reproductive care.
